# Association of Allelic Variation in *PtoXET16A* with Growth and Wood Properties in *Populus tomentosa*

**DOI:** 10.3390/ijms150916949

**Published:** 2014-09-23

**Authors:** Bowen Wang, Deqiang Zhang

**Affiliations:** 1 National Engineering Laboratory for Tree Breeding, College of Biological Sciences and Technology, Beijing Forestry University, Beijing 100083, China; E-Mail: 15210908561@yeah.net; 2 Key Laboratory of Genetics and Breeding in Forest Trees and Ornamental Plants, Ministry of Education, College of Biological Sciences and Technology, Beijing Forestry University, Beijing 100083, China

**Keywords:** xyloglucan *endo*-transglycosylase, single-nucleotide polymorphism, intron splicing site variation, linkage disequilibrium, *Populus tomentosa*

## Abstract

Xyloglucan *endo*-transglycosylases (XETs) modify the xyloglucan-cellulose framework of plant cell walls and, thus, affect cell wall expansion and strength. Dissecting the mechanism by which natural variation in XETs affects wood properties can inform breeding efforts to improve wood quality and yield traits. To this end, we isolated a full-length *PtoXET16A* cDNA clone from *Populus tomentosa*. Real-time PCR analysis showed that *PtoXET16A* was maximally expressed in the root, followed by phloem, cambium, and developing xylem, suggesting that PtoXET16A plays important roles in the development of vascular tissues. Nucleotide diversity and linkage disequilibrium analysis revealed that *PtoXET16A* has high single nucleotide polymorphism (SNP) diversity (π = 0.01266 and θ_w_ = 0.01392) and low linkage disequilibrium (*r*^2^ ≥ 0.1, within 900 bp). SNP- and haplotype-based association analyses of 426 individuals from a natural population indicated that nine SNPs (including two non-synonymous markers and one splicing variant) (*p* ≤ 0.05, false discovery rate *Q* ≤ 0.01), and nine haplotypes (*p* ≤ 0.05) were significantly associated with growth and wood properties, each explaining from 3.40%–10.95% of phenotypic variance. This work shows that examination of allelic variation and linkage disequilibrium by a candidate-gene-based approach can help to decipher the genetic basis of wood formation. Moreover, the SNP markers identified in this study can potentially be applied for marker-assisted selection to improve growth and wood-property traits in *Populus*.

## 1. Introduction

Woody tissues are composed of various biopolymers and provide an enormous, renewable feedstock for pulp and paper, biofuels, and solid wood products [1]. Forest tree-breeding programs aim to improve the quantity and quality of wood products from trees grown in plantations. Considering that small improvements in quantitative traits may deliver large gains, identification of genes and gene variants controlling growth and wood quality traits can provide important information for forest tree-breeding programs. Xyloglucan *endo*-transglycosylases (XETs), among the most important enzymes affecting cell wall expansion and strength, modify the xyloglucan-cellulose framework of plant cell walls [2]. XETs break the β-(1→4) glycosidic bond in the xyloglucan backbone and transfer the xyloglucanyl segment to O-4 of the non-reducing terminal glucose residue of an acceptor, which may be either xyloglucan or a xyloglucan-oligosaccharide [3,4]. The *XETs* make up part of a multigene family of xyloglucan *endo*-transglycosylases/hydrolases (*XTHs*) [5]. *XTH* family evolution can be visualized by analysis of the 17 publicly available land plant genomes (http://www.phytozome.org/), and this analysis suggests that higher plants have large *XTH* gene families [6]. For example, previous studies showed there are 33 *XTH* members in *Arabidopsis thaliana* [7], 29 in rice (*Oryza sativa*) [8] and 41 in *Populus trichocarpa* [9]. The *XTHs* have three main subfamilies according to the structure of protein, and enzymes encoded by the *XTH* genes in subfamilies I and II show XET activity [10]. By contrast, enzymes encoded by the *XTH* genes in subfamily III-A have a short conserved sequence in the catalytic domain, and show xyloglucan *endo*-hydrolase (XEH) activity, but III-Bs show XET activity [11].

XETs are thought to play a major role in the regulation of cell wall stress relaxation and gravitropic responses, and in the incorporation of nascent xyloglucan into the wall during biosynthesis. In previous studies, at least 16 *Populus* *XTH* genes, all likely encoding XETs, were expressed in developing wood [5]. Wood tissues that have ceased growing show detectable XET activity [12,13]. In addition, PttXET16A from the hybrid aspen *Populus tremula × tremuloides* plays role in restructuring primary walls during the deposition of secondary wall layers, probably by creating and reinforcing the connections between the primary and secondary wall layers [14], implying that XETs play a role in carbohydrate transglycosylation within and between different cell wall layers of xylem cells [15]. *XETs* are also actively transcribed in tissue-, time-, and stimulus-dependent contexts. *AtXTH31* modulates XET activity in roots, thus possibly regulating the content of xyloglucan. In *Arabidopsis*, xyloglucan can bind aluminum, which accumulates in the cell wall [16]. Furthermore, various growth-promoting hormones up-regulate XETs, including gibberellin acid (GA), auxin and brassinolide [17,18,19]. For example, in soybean, brassinosteroids enhance expression of *BRU-1*, which encodes an XET that could have a role in increasing the plastic extensibility of epicotyl segments. XET isoforms reportedly induce either strengthening or loosening of heat-inactivated cell walls [20]. Individual XET isoforms may act as either wall-loosening or wall-strengthening agents, depending on the isoform, but no isoform-specific properties have been identified that could explain the differences in their effects [21,22,23,24].

Wood quality and yield are key traits for cultivation of trees and many wood- and yield-related traits vary quantitatively; to improve these traits, researchers have shown tremendous interest in using association mapping to identify the genes responsible for this quantitative variation. Association mapping identifies quantitative trait loci by examining the marker-trait associations that can be attributed to the strength of linkage disequilibrium (LD) between markers and functional polymorphisms across a set of diverse germplasm. Combined with exploiting natural diversity and development of robust statistical analysis methods, association mapping is becoming one of the main methods for dissecting the genetic architecture of key traits and has been widely used in forest tree species [25,26,27]. Of these tree species, *Populus* *tomentosa*, as one of the main commercial tree species used for timber production in China, plays an indispensable role in ecological and environmental protection along the Yellow River. Therefore, association studies of single nucleotide polymorphisms (SNPs) associated with growth and wood properties of *P. tomentosa* are essential to detect functional allelic variation for marker-assisted selection in breeding programs that aim to improve the quality and quantity of wood products in* P*. *tomentosa*.

Here, we used a candidate gene based approach to examine genetic variation in the *P. tomentosa XET* homolog *PtoXET16A*. We used a combination of single-marker- and haplotype-based association methods in an association population to identify several associations underlying natural variation of complex growth and wood properties. Our study provides a necessary foundation for improving the quantity and quality of wood by breeding in the tree crop species *P. tomentosa*.

## 2. Results

### 2.1. Isolation of PtoXET16A from P. tomentosa

**Figure 1:**
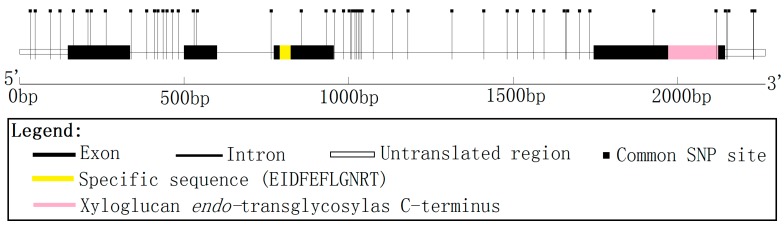
Genomic organization of *PtoXET16A*. Exons are shown as boxes and introns as lines. Positions of common SNP markers are shown as vertical lines. The active site of glycosyl hydrolases family 16 EIDFEFLGNRT (at residues 107–117) and a xyloglucan *endo*-transglycosylase (XET) *C*-terminus in fourth exon (at residues 234–284), identified by analysis of protein sequence for Pfam matches (http://pfam.sanger.ac.uk/), are shown.

A full-length cDNA encoding a XET16A-like protein was isolated from a cDNA library prepared from mature xylem zone of *P. tomentosa*. The cDNA clone (GenBank Accession No. KM267530) is 1141 bp in length, and contains a full-length open reading frame (870 bp), encoding a polypeptide of 290 amino acids with an estimated molecular mass of 33.70 kD and a pI of 7.62 (http://web.expasy.org/protparam/), flanked by 146 bp of 5' untranslated region (5'UTR) and 122 bp of 3'UTR (Figure 1). Alignment of the full-length cDNA sequence to the genomic sequence showed that *PtoXET16A* has three introns and four exons (Figure 1). Identification of protein domains, families and functional sites by matches to the Prosite database (http://prosite.expasy.org/prosite.html) and analysis of the protein sequence for Pfam matches (http://pfam.sanger.ac.uk/) showed that the predicted protein has the active site of glycosyl hydrolase family 16 EIDFEFLGNRT (at residues 107–117) (Figure 1) and an XET *C*-terminal sequence in the fourth exon (at residues 234–284) (Figure 1).

**Figure 2:**
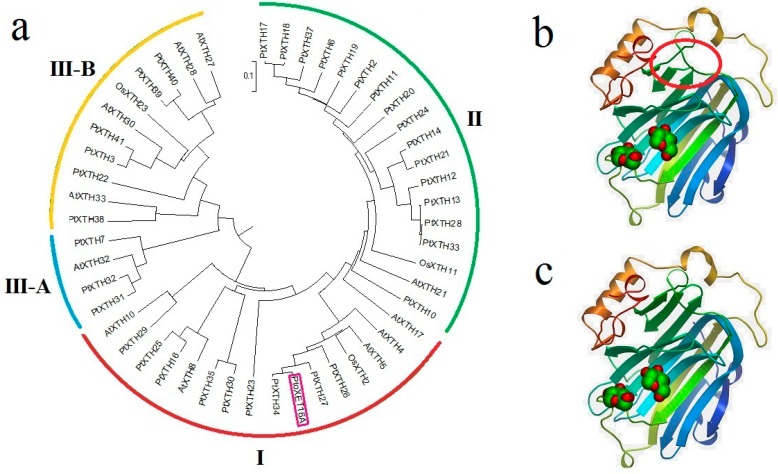
A rooted phylogenetic tree and three-dimensional structures of *XTH* gene products. (**a**) A rooted phylogenetic tree of PtoXET16A and other predicted products of *XTH* genes. The similarity to other *XTH* gene products was calculated using the UPGMA program. Full-length protein sequences were used for the comparison and the gene models used are listed in Table S1. The phylogenetic tree presents predicted protein sequences for the *XTH* family of *P. trichocarpa*, numbered according to Geisler-Lee *et al.* [9], *Arabidopsi*s* thaliana* XTH proteins, numbered according to Yokoyama and Nishitani [7], and *Oryza sativa* *XTH* gene products, numbered according to Yokoyama *et al.* [8]; (**b**) Three-dimensional structures of PtoXET16A constructed using Swissmodel (http://swissmodel.expasy.org/); (**c**) Three-dimensional structures of PttXET16-34, constructed using Swissmodel (http://swissmodel.expasy.org/). The polypeptide chain is colored from blue (*N* terminus) to red (*C* terminus). The red circle shows the location of four missing amino acids (YIIV) compared with PttXET16-34.

The molecular phylogeny of *XTH* gene products includes three major branches (I/II, IIIA and IIIB) (Figure 2). Of these, the largest cluster confirmed previous studies that suggested merging groups I and II. This analysis indicates that *PtoXET16A* belongs to group I. A BLASTP search with PtoXET16A as the query sequence revealed that the PtoXET16A protein shares 98% identity with PttXET16-34 (AAN87142), 79% identity with AtXTH5 (AT5G13870) and 76% with OsXTH2 (Os11g0539200) (Figure 2, Table S1). The alignment shows that PtoXET16A lacks four amino acids (YIIV) that are present in the XET16As from other species. The tertiary structure predicted using Swissmodel (http://swissmodel.expasy.org/), showed that PtoXET16A and PttXET16-34 have similar structures. However, the amino acids missing in PtoXET16A but present in PttXET16-34 did produce a structural difference in one region (Figure 2).

### 2.2. Analysis of PtoXET16A Expression

**Figure 3:**
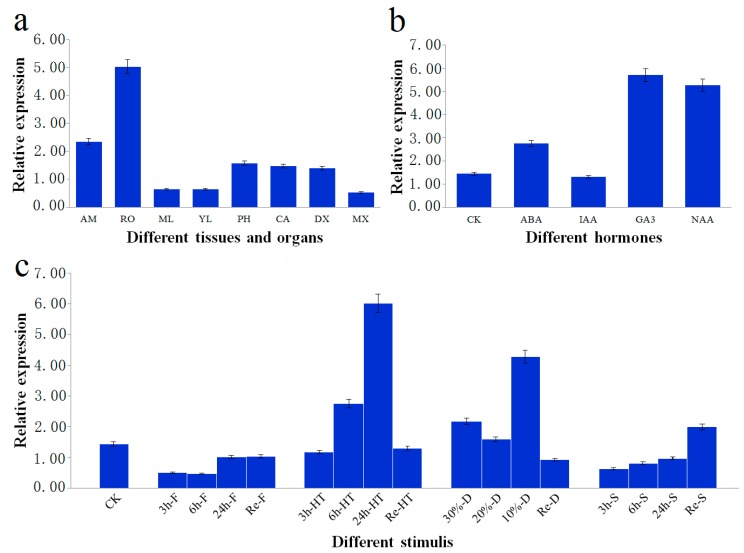
Relative transcript levels of *PtoXET16A*. The error bars represent ± standard deviation. (**a**) Relative transcript levels of *PtoXET16A* in *P. tomentosa* tissues and organs. *AM*, apical meristem; *RO*, root; *ML*, mature leaves; *YL*, young leaves; *PH*, phloem; *CA*, cambium; *DX*, developing xylem; *MX*, mature xylem; (**b**) Relative transcript levels of *PtoXET16A* before and after different treatments in *P. tomentosa*. *CK*, control check; *ABA*, abscisic acid; *IAA*, indoleacetic acid;* GA*, gibberellin acid; *NAA*, naphthylacetic acetic acid. (**c**) Expression analysis in* P. tomentosa* of *PtoXET16A* in response to abiotic stresses; *CK*, control check; *Re*, recovered condition; *F*, freezing stress; *HT*, high-temperature stress; *D*, drought condition; *S*, high-salinity stress. Samples were exposed to 150 mM NaCl, 4 °C and 42 °C for 3, 6, and 24 h for high-salinity, freezing stress and high temperature stress treatments, respectively. Drought condition was induced by withholding soil water content to 30%, 20%, and 10% of their original content at room temperature. The treated plants were then transferred to pots under normal growing conditions for 24 h to recover from cold, heat, drought and high salinity, which were denoted as Re-F, Re-HT, Re-D, Re-S, respectively. As control, samples without treatments were used.

We determined to what extent *PtoXET16A* exhibits tissue-specific expression in *P*. *tomentosa*. Levels of *PtoXET16A* mRNA in various poplar tissues, including apical meristem, root, phloem, cambium, developing xylem, mature xylem, young leaf and mature leaf, were measured by quantitative real time-PCR (RT-PCR) with gene-specific primers and *Actin* as an internal control (Figure 3a). *PtoXET16A* mRNA was the most abundant in root (5.033 ± 0.012), followed by phloem (1.573 ± 0.002), cambium (1.471 ± 0.009), and developing xylem (1.392 ± 0.006). In contrast, relatively lower abundances of *PtoXET16A* mRNA were detected in mature leaf (0.647 ± 0.013), young leaf (0.637 ± 0.002) and mature xylem (0.530 ± 0.016). These observations indicated that *PtoXET16A* shows preferential expression in vascular tissues, suggesting that *PtoXET16A* plays an important role in wood formation.

We further tested whether hormone treatments induced *PtoXET16A* expression, testing abscisic acid (ABA), indoleacetic acid (IAA), GA, and naphthylacetic acetic acid (NAA) (Figure 3b) in *P*.* tomentosa*. The results revealed that the expression of *PtoXET16A* was induced by treatment with most plant hormones, except for IAA (Figure 3b). Of these treatments, the expression level of *PtoXET16A* following GA treatment was more than four times higher than the controls, indicating that the expression of *PtoXET16A* could be strongly regulated by GA.

We further tested whether *PtoXET16A* was inducible by different abiotic stresses (Figure 3c). Similar expression patterns were observed in plants exposed to freezing, heat and high-salinity stresses, in which the relative *PtoXET16A* mRNA levels gradually increased over the course of the stress treatment (Figure 3c). Compared with the control, the relative expression of *PtoXET16A* was repressed in freezing and high-salinity stresses; conversely, *PtoXET16A* expression was significantly induced in heat and drought conditions. When the plants recovered, *PtoXET16A* expression returned to the level of the control (Figure 3c). We also found that the strongest relative expression of *PtoXET16A* was in response to high-temperature stress (Figure 3c). Under drought conditions, the highest expression was observed at 10% soil water content (4.260 ± 0.011), followed by 30% soil water content (2.171 ± 0.007) (Figure 3c). These results indicated that *PtoXET16A* expression is sensitive to heat and drought stimuli.

### 2.3. SNP Diversity and Genotyping

To identify SNPs in *PtoXET16A*, the approximately 2266 bp genomic region of *PtoXET16A* was amplified and sequenced from 43 unrelated individuals, representing almost the entire natural range of *P*. *tomentosa*. Table 1 summarizes the statistical analysis of nucleotide polymorphisms over different regions of *PtoXET16A*. Across the samples, 134 SNPs were detected in the whole gene at a frequency of approximately one SNP every 17 bp (Table 1). Forty-three of these SNPs occurred in exons, and included 18 missense and 25 nonsense mutations (Table 1). All together, 49 of 134 SNPs (38.1%) were considered as common (frequency > 0.10). In general, the *PtoXET16A* locus has high nucleotide diversity (π), with π = 0.01266 and θ_w_ = 0.01392, respectively (Table 1). More specifically, estimates of nucleotide diversity, π, for the different gene regions ranged from 0.00239 (intron 2) to 0.02461 (intron 1), and θ_w_ varied between 0.00142 (intron 2) and 0.01985 (intron 1). Within coding regions, the non-synonymous nucleotide substitution rate (π_nonsyn_) was markedly lower than π_syn_, with a π_nonsyn_/π_syn_ ratio of 0.2554 < 1.0, suggesting that diversity at the synonymous sites of exon regions resulted from strong purifying selection (Table 1). The 49 common SNPs were successfully genotyped across 426 trees in the association population by using locked nucleic acid technology.

Genetic differentiation within and among three geographically independent climatic regions was examined using the nucleotide diversity data from *PtoXET16A* (Table 2). Levels of nucleotide variation (measured using π) in the three climatic regions varied, but showed similar patterns of π_tot_, π_sil_, π_s_ and π_n_ (Table 2). These observations suggested that the level of selective constraint was similar between the three climatic regions. Tajima’s D was positive in the southern, northeastern and northwestern climatic regions but negative in the *P. tomentosa* population as a whole; however, no significant departures from the neutral expectation were observed (Table 2). The Fu and Li’s D statistical tests were positive for the northeastern and northwestern populations, but were negative for the southern region and the* P. tomentosa* population as a whole, revealing the existence of an excess of low-frequency mutations for this gene region in the *P. tomentosa* species-wide samples (Table 2).

**Table 1:** Nucleotide polymorphisms at the *PtoXET16A* locus.

Region	No. of bp	No. of Polymorphic Sites	Percentage Polymorphism	Nucleotide Diversity	
				π	θ_w_
5'UTR	146	5	3.42	0.01350	0.00792
Exon 1	190	12	6.32	0.01226	0.01460
Synonymous	45.10	3	6.65	0.01290	0.01537
Non-synonymous	143.9	9	6.25	0.01216	0.01446
Intron 1	163	14	8.59	0.02461	0.01985
Exon 2	101	4	3.96	0.00947	0.00915
Synonymous	23.00	4	17.39	0.04160	0.04019
Non-synonymous	76.00	0	0.00	0.00000	0.00000
Intron 2	173	1	0.58	0.00239	0.00142
Exon 3	182	2	1.10	0.00433	0.00254
Synonymous	38.28	1	2.61	0.00885	0.00604
Non-synonymous	141.72	1	0.71	0.00317	0.00163
Intron 3	789	61	7.73	0.01746	0.01851
Exon 4	400	25	6.25	0.00548	0.01445
Synonymous	83.60	10	11.96	0.01682	0.02765
Non-synonymous	312.40	15	4.80	0.00252	0.01110
3'UTR	122	10	8.20	0.01810	0.01894
Total	2266	134	5.91	0.01266	0.01392
Synonymous	190.98	18	9.43	0.01719	0.02178
Non-synonymous	679.02	25	3.68	0.00439	0.00851

Regions containing indels were excluded from the calculation.

**Table 2:** Summary of nucleotide variation in *PtoXET16A* in *P*.* tomentosa* natural populations from three climatic regions.

Population	*N*	π_tot_	π_sil_	π_s_	π_n_	Tajima’s D	Fu and Li’s D
Northeastern region	14	0.01380	0.01786	0.01747	0.00447	0.95307	0.51753
Southern region	15	0.01309	0.01657	0.01814	0.00505	0.71556	−0.31283
Northwestern region	14	0.01166	0.01509	0.01670	0.00377	0.48336	0.02633
Total	43	0.01266	0.01625	0.01719	0.00439	−0.33198	−2.48533

*N*, number of sequences sampled; π_tot_, average nucleotide diversity in full gene; π_sil_, average nucleotide diversity in synonymous and noncoding sites; π_s_, average nucleotide diversity of synonymous mutation; π_n_, average nucleotide diversity of non-synonymous mutation.

### 2.4. Linkage Disequilibrium and Phenotype-Genotype Associations

**Figure 4:**
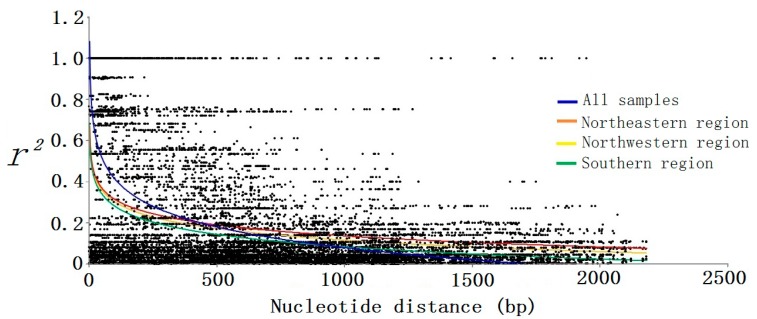
The decay of short-range linkage disequilibrium within* PtoXET16A* for all samples and each climatic region. We sequenced the *PtoXET16A* regions from a panel of 43 unrelated individuals (15 from the southern region, 14 from the northwestern region, and 14 from the northeastern region). Pairwise correlations between SNPs are plotted against the physical distance between the SNPs in base pairs. The curves describe the nonlinear regression of *r*^2^ onto the physical distance in base pairs.

The nonlinear regression shows a clear and rapid decline of LD with distance in base pairs within *PtoXET16A* (*r*^2^ ≥ 0.1, within 900 bp), indicating that LD of the SNP loci did not extend over the entire gene region (Figure 4). Within-group analyses of LD showed a similar decline in samples from the southern region, with the *r*^2^ values declining to 0.1 within 900 bp. Nevertheless, we observed a higher level of LD within samples from the northeastern and northwestern regions, with the *r*^2^ values declining to 0.1 within approximately 1700 bp. These results revealed that the northeastern and northwestern regions seem to have experienced similar histories and the southern region had a higher evolutionary rate. Associations between 30 SNPs and 10 growth and wood quality traits were tested by using the mixed linear model (MLM) in TASSEL version 2.1 (Buckler lab, New York, NY, USA, 2010). The MLM identified 37 significant markers (*p* < 0.05), but correction for false discovery rate (FDR) (*FDR* < 0.05) reduced this to 13. These associations were identified in the exon, intron, and 3'UTR regions of *PtoXET16A* (Table 3).

**Table 3:** SNP markers significantly associated with growth and wood properties in the association population.

Trait	Marker	Position	Mutation	*p*-Value	*FDR*	*r*^2^ (%)
Lignin content	SNP6	Exon 1	[G:C] ^ns^	<0.001	<0.001	10.95
	SNP16	Intron 3	[C:T]	<0.001	0.009	5.37
	SNP29	3'UTR	[G:C]	<0.001	0.003	6.16
*D*	SNP15	Exon 3	[C:T] ^ns^	<0.001	0.012	4.27
*V*	SNP15	Exon 3	[C:T] ^ns^	<0.001	0.012	4.25
Fiber length	SNP21	Intron 3	[C:T]	<0.001	0.001	5.70
	SNP22	Intron 3	[G:T]	<0.001	0.001	5.66
	SNP23	Intron 3	[C:T]	<0.001	0.020	3.77
	SNP29	3'UTR	[G:C]	0.001	0.031	3.40
Fiber width	SNP27	Exon 4	[A:G] ^s^	0.001	0.031	3.65
*MFA*	SNP14	Intron 2	[C:T]	0.001	0.031	3.56
	SNP21	Intron 3	[C:T]	0.002	0.038	3.40

*D*, diameter at breast height; *V*, stem volume; *MFA*, microfiber angle; ns, non-synonymous polymorphism; *s*, synonymous polymorphism; *p*-value, the significant level for association (the significance is *p* ≤ 0.05); *r*^2^, percentage of the phenotypic variance explained; *FDR*, false discovery rate (the significance is* FDR* ≤ 0.05).

#### 2.4.1. Single-SNP-Based Association

In total, twelve associations representing nine significant SNPs were identified (Table 3). Of these, SNP15, a missense mutation in exon 3, resulted in an encoded amino acid change from Tyr to His, and was significantly associated with two traits, including diameter at breast height and stem volume (Table 3). SNP6, another missense mutation in exon 1, resulted in an encoded amino acid change from Ala to Pro, was significantly associated with lignin content and explained 10.95% of the phenotypic variance (Table 3). SNP21 in intron 3, a synonymous mutation, was found to be associated with fiber length and microfiber angle, and SNP29 from the 3'UTR showed genetic association with fiber length and lignin content (Table 3). Markedly, SNP16, one splice variation in the second base of intron 3, resulted in splice junction (splice donor) from GT to GC, was significantly associated with lignin content and explained 5.37% of the phenotypic variance. All together, these SNP loci explained a small proportion of the phenotypic variance, with the individual effects ranging from 3.40% to 10.95% (Table 3). These small SNP effects are in accordance with polygenic quantitative models of complex traits.

**Figure 5:**
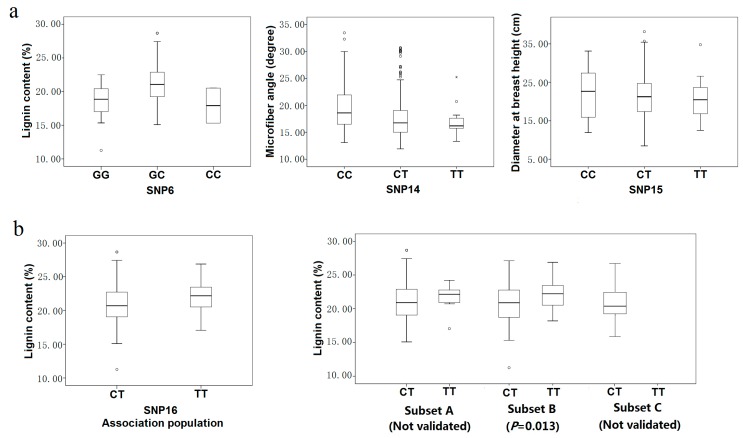
Genotypic effect on SNP markers in the association population (**a**) Three modes of gene action quantified using the ratio of dominant to additive effects estimated from least-square means for each genotypic class. Three modes of gene action were observed as over-dominance (SNP6), full dominance (SNP14) and co-dominance (SNP15); (**b**) Genotypic effect on SNP6 with lignin content in the discovery population and three subsets (Subset A, 170 individuals from the southern region; Subset B, 91 individuals from the northwestern region; Subset C, 165 from the northeastern region).

Most of the associations were consistent with modes of gene action other than codominance (Table 4). Five of the 12 marker-trait pairs for which dominance and additive effects could be calculated were consistent with over- or underdominance (|d/a| > 1.25). For example, heterozygotes for SNP6 had higher lignin content, on average, than either homozygote class (18.09% for GG, 21.02% for GC, 17.92% for CC) (Figure 5a). The remaining seven marker-trait pairs were split between modes of gene action that were partially to fully dominant (0.50 < |d/a| < 1.25) (Figure 5a), such as SNP14 (20.96 for CC, 17.59 for CT, 17.26 for TT in microfiber angle) or codominant (|d/a| ≤ 0.5), such as SNP15 (22.14 for CC, 21.44 for CT, 21.14 for TT in diameter at breast height) (Figure 5a).

**Table 4:** List of marker effects for significant marker-trait pairs.

Trait	SNP	2a ^a^	d ^b^	d/a	2a/s_p_^c^	Frequency ^d^		a ^e^
Lignin content	SNP6	1.333	2.43	3.646	0.531	0.482	C	−1.412
	SNP16	22.019	9.7645	0.887	8.353	0.461	C	3.524
	SNP29	5.909	−2.8905	−0.978	2.241	0.335	G	1.964
*D*	SNP15	0.994	−0.211	−0.425	0.177	0.485	T	−0.164
*V*	SNP15	0.029	−0.0255	−1.759	0.075	0.485	T	0.003
Fiber length	SNP21	0.0214	0.0153	1.43	0.255	0.495	T	−0.012
	SNP22	0.087	−0.0225	−0.517	1.036	0.494	G	0.032
	SNP23	0.013	−0.002	−0.308	0.163	0.460	C	−0.003
	SNP29	0.152	0.081	1.066	1.81	0.335	G	−0.051
Fiber width	SNP27	2.189	2.2155	2.024	1.104	0.496	A	−1.624
*MFA*	SNP14	3.703	−1.5205	−0.821	0.818	0.479	T	−0.385
	SNP21	1.414	−1.661	−2.349	0.313	0.495	T	0.340

*D*, diameter at breast height; *V*, stem volume; *MFA*, Microfiber angle; ^a^ Calculated as the difference between the phenotypic means observed within each homozygous class (2a = |G_BB_ − G_bb_|, where G_ij_ is the trait mean in the ijth genotypic class); ^b^ Calculated as the difference between the phenotypic mean observed within the heterozygous class and the average phenotypic mean across both homozygous classes [d = G_Bb_ − 0.5(G_BB_ + G_bb_), where G_ij_ is the trait mean in the ijth genotypic class]; ^c^ s_p_, standard deviation for the phenotypic trait under consideration; ^d^ Allele frequency of either the derived or minor allele. Single nucleotide polymorphism (SNP) alleles corresponding to the frequency listed are given in parentheses; ^e^ The additive effect was calculated as a = p_B_(G_BB_) + p_b_(G_Bb_) − G, where G is the overall trait mean, G_ij_ is the trait mean in the ijth genotypic class and p_i_ is the frequency of the ith marker allele. These values were always calculated with respect to the minor allele.

#### 2.4.2. Haplotype-Based Associations

**Figure 6:**
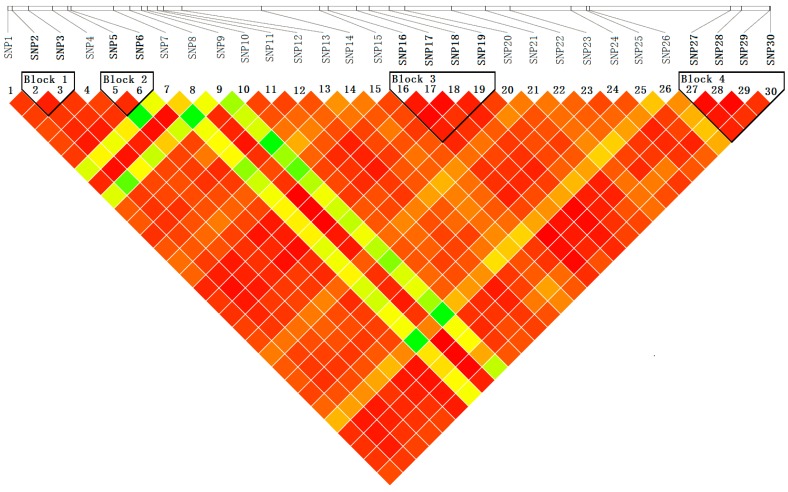
Pairwise linkage disequilibrium (*r*^2^) between SNP markers. The common genotyped SNPs are shown on a schematic of *PtoXET16A* and the pairwise *r*^2^ values are shown by color-coding in the matrix below.

Haplotype analysis by Haploview, using genotype data for 30 SNPs from 426 individuals in the association population, showed four distinct haplotype blocks within *PtoXET16A* (Figure 6). We used a haplotype trend regression test [28] to identify the haplotypes significantly associated with the 10 growth and wood quality traits. Three common haplotypes (allele frequency > 5%, *p*-value < 0.05) were observed with significant effect on these traits. These haplotypes span exon 1, intron 3, exon 4 and the 3'UTR (Figure 6). The proportion of phenotypic variation explained by these haplotypes ranged from 1.63% to 10.46% (Table 5). Among them, three haplotypes from SNP5-6 and SNP16-19 were associated with diameter at breast height, with the individual effects ranging from 1.63% to 2.71%. In addition, association between the three haplotypes and stem volume were observed, which explained 3.05%–4.41% of the phenotypic variance (Table 5). Of these, two haplotypes from SNP5-6 were associated with lignin content and one haplotype from SNP27-30 was associated with microfiber angle (Table 5).

**Table 5:** Haplotypes significantly associated with growth and wood property traits.

Trait	*p* (Overall)	*r*^2^ (%)	Haplotype	Frequency	Mean	*p* (ind)
Diameter at breast height	0.0235	1.63	SNP5-6			
			G-C	0.0666	19.7014	0.010
			T-C	0.4513	21.6812	0.010
	<0.0001	2.71	SNP16-19			
			C-T-A-T	0.4062	21.0053	<0.001
Stem volume	0.0005	3.05	SNP5-6			
			G-C	0.0666	0.4210	<0.001
			T-C	0.4501	0.6243	<0.001
	0.0038	4.41	SNP16-19			
			C-T-A-T	0.4062	0.5638	<0.001
Lignin content	0.0013	10.46	SNP5-6			
			G-C	0.0687	19.8953	0.003
			G-G	0.4744	20.9963	<0.001
Microfiber angle	0.4737	3.86	SNP27-30			
			G-A-C-T	0.4871	15.235	0.050

*p*-value, the significant level for haplotype-based association (the significance is *p* ≤ 0.05); *r*^2^, percentage of the phenotypic variance explained.

#### 2.4.3. Validation of Association Testing

Three smaller subsets derived from the association population were used to validate the significant single-SNP associations identified in the association population (Table 6). Eight of the twelve significant marker-trait associations were validated in at least one of the three smaller subsets. In total, six SNP markers (SNP6, SNP14, SNP15, SNP16, SNP21, and SNP29) were significantly associated with five traits, including fiber length, lignin content, *D*, *V* and *MFA* at the threshold of *p* < 0.05. The proportion of phenotypic variation explained by the six SNP markers varied from 2.39% to 13.07% (Table 6). Associations of SNP22, SNP23 and SNP27 with fiber length/width were not validated in any of the three smaller subsets. The failure to validate these three significant associations in this study may arise from the complexity of quantitative traits, the small sample size or other factors.

### 2.5. Transcript Analysis of SNP Genotypes

**Figure 7:**
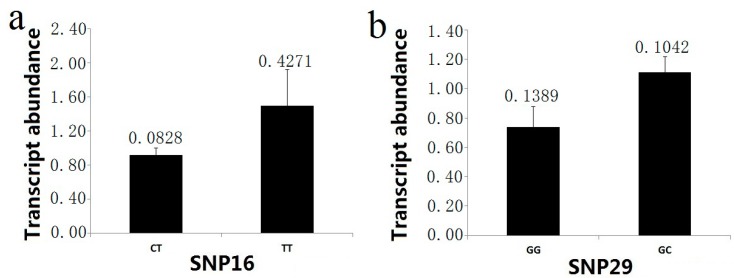
*PtoXET16A* transcript abundance varies among genotypic classes for significant SNP associations. The error bars represent ± standard deviation. (**a**) Transcript abundance in the two genotypic classes for SNP16; (**b**) Transcript abundance in the two genotypic classes for SNP29.

To identify whether these significant associations affect gene expression at the mRNA level, we validated SNP associations via gene expression analyses. Transcript levels among the different genotypic classes for nine significantly associated SNPs (Table 3) were compared by RT-PCR with gene-specific primers. The assays used secondary xylem from the 20-year-old trees to quantify the mRNA levels in 30 trees (including almost all genotypes). Measurement of different transcript abundance across three (using analysis of variance, ANOVA) or two genotypic classes (using *t*-test) indicated that two markers (SNP16 and SNP29) showed significant differences in the RNA transcript levels in the association population. For the marker SNP16 (intron 3, IVS3 + 2T>C), the higher abundance of the mRNA (1.4922 ± 0.4271) was found in the TT group, and the transcript level of the CT group was 0.9175 ± 0.0828 (Figure 7). In examining genotype-specific transcript levels for SNP29 (3'UTR), the heterozygous trees (1.1116 ± 0.1042 in GC group) for this marker showed higher relative mRNA abundance than the homozygous trees (0.7390 ± 0.1389 in GG group) (Figure 7).

**Table 6:** SNP markers significantly associated with growth and wood properties traits in three subsets.

Trait	Marker	Subset A		Subset B		Subset C	
		*p*-Value	*r*^2^ (%)	*p*-Value	*r*^2^ (%)	*p*-Value	*r*^2^ (%)
Lignin content	SNP6	\	\	≤0.001	10.35	≤0.001	2.39
	SNP16	\	\	0.013	3.81	\	\
*D*	SNP15	\	\	0.008	5.69	\	\
*V*	SNP15	\	\	0.008	5.69	\	\
Fiber length	SNP21	\	\	0.009	5.55	\	\
	SNP29	0.009	4.19	0.020	4.64	\	\
*MFA*	SNP14	\	\	0.047	3.69	0.045	6.82
	SNP21	\	\	≤0.001	13.07	\	\

*D*, diameter at breast height; *V*, stem volume; *MFA*, microfiber angle; *p*-value, the significant level for association (the significance is *p* ≤ 0.05); *r*^2^, percentage of the phenotypic variance explained; Subset A, northwestern region; Subset B, southern region; Subset C, northeastern region.

## 3. Discussion

### 3.1. Nucleotide Diversity and LD in PtoXET16A

SNP-based association mapping requires a comprehensive investigation of the patterns of SNP distribution and frequency within the full-length *PtoXET16A* locus in a natural population of *P. tomentosa*. Within coding regions, the π_nonsyn_:π_syn_ ratio (0.2554) was significantly less than 1 for *PtoXET16A*, as commonly observed for other genes in natural populations of forest trees [29]. Synonymous mutations occurring during evolution may be fixed with a higher probability than neutral ones because of purifying selection. Interestingly, levels of average nucleotide diversity in noncoding regions were lower than in the coding regions (Table 1) due to conservation of intron 2, in which just one SNP locus (SNP14) was observed. Some introns experience higher selective constraints than synonymous coding sites, possibly because they harbor key regions for regulation of expression [30]. This suggests that these conserved intron sequences may affect gene regulation [31]. In this study, a significantly higher frequency of polymorphisms was found in intron 1 than in the other regions in *PtoXET16A* (π = 0.02461) (Table 1). This finding suggests that this noncoding region may be relatively unstable and a “hotspot” for genetic change [32].

LD describes a key aspect of genetic variation in natural populations. We examined LD across tree climatic regions and observed a rapid decay of LD within just a few hundred base pairs, indicating that association genetics has the potential to identify the genes responsible for variation in these traits. Previous studies in *P. tremula* (five genes) [33], *P. nigra* (nine genes) [34] and *P. trichocarpa* (39 genes) [35] showed a similarly rapid decay of LD. In this study, the level of LD decay in *PtoXET16A* was analyzed separately within each of the three climatic regions and for the complete natural population (Figure 4). The results showed that the northeastern region had higher LD than the northwestern and southern regions, consistent with the higher frequency of exclusive SNPs observed in this region (Table 2). Our results show that the LD in *PtoXET16A* within three climatic regions may be more extensive than the LD found in our range-wide *P. tomentosa* samples (Figure 4), consistent with previous studies [36,37]. However, a recent genome-wide study of the extensive LD in *P. trichocarpa* (*r*^2^ > 0.2, within 3–6 kb) suggests that genome-wide association studies and genomic selection in natural populations may be more feasible in *Populus* than previously assumed [38]. Therefore, our future work will focus on estimation of LD decay with greater genomic coverage and exploration of the variability of haplotype structure across the entire genome. Such studies will also help to elucidate how *Populus* managed to adapt to a wide variety of environmental conditions [39].

### 3.2. The Putative Functional Roles of XETs

Xyloglucan is incorporated and modified in the cell wall network by various enzymes encoded by *XTHs*, and forty-one *XTH* gene models can be found in the *P. trichocarpa* genome. Genome sequencing projects have revealed the presence of *XTHs* in various plant species and their transcription in various plant tissues [10,40,41]. For example, *PtXTH10* and *PtXTH24* are expressed in shoot tips, young leaves, mature leaves, and roots [42]. *OsXTH8* is expressed in rice leaf sheath, root, leaf blade and callus [18]. *AtXTH4* and *AtXTH27* are highly and ubiquitously expressed in most organs including leaf, stem, flower and silique [7]. We found *PtoXET16A* expression in most organs and tissues, with the most abundant expression in root, followed by phloem, cambium and developing xylem (Figure 3a). These results are consistent with studies of its ortholog *AtXTH5*, which exhibits root-specific expression [7]. These results also support the finding that *XTH* genes expressed in the cambial region are also strongly expressed in the developing phloem [5].

The existence of at least 16 *XET* genes in *Populus* suggests that individual *XETs* may exhibit distinct patterns of expression in different developmental stages and in response to hormonal and environmental stimuli [5,15,40]. The expression of *PtXTH22* shows up-regulation in response to various hormones including 6-benzylaminopurine, IAA, salicylic acid, GA, brassinosteroids, jasmonic acid, and ABA [42]. In our study, the expression of *PtoXET16A* was up-regulated by most of the plant hormone treatments, except for IAA (Figure 3b). Of these, *PtoXET16A* was most strongly induced by GA, to four times higher mRNA levels than the controls (Figure 3b). Transcriptional up-regulation of *XTH* expression by GA has been demonstrated in several instances [43]. For example, *OsXTH8* expression increased in a dose- and time-dependent manner with GA_3_ treatment [18]. In* P. tomentosa* (Figure 3b), similar expression patterns were also observed, indicating a general interaction between XETs and GA. Products of *XET*-encoding genes may be responsible for the resulting cell wall changes in response to plant hormones. As signals that mediate plant systemic responses [44], interactions between plant hormones also modulate the expression of stress-responsive genes [45]. Hence, it is expected that *PtoXET16A* is up-regulated in response to abiotic stresses in* P*.* tomentosa* (Figure 3c). The relative expression levels increased gradually over time in response to freezing, heat and high-salinity stresses (Figure 3c). After recovery, *PtoXET16A* expression returned to the level of control plants (Figure 3c), suggesting that *PtoXET16A* maintained a certain level of expression under all four stimuli. Because the cell wall is a primary determinant of cell and organ shape, alterations in morphogenesis would most likely require cell wall modification. Since XETs modify a major component of the plant cell wall, they may have important functions in altering wall properties in response to environmental conditions. Moreover, the level was significantly up-regulated under heat and drought conditions, indicating that *PtoXET16A* expression is sensitive to heat and drought stimuli [19,46]. XETs have been detected in plants responding to stresses including flooding [47], salt tolerance [46] and others.

### 3.3. Dissecting Allelic Polymorphisms Underlying Growth and Wood Properties

*XETs* encode key enzymes acting in the formation and modification of the carbohydrate matrix of wood cell walls, justifying selection of an XET as a targeted candidate gene for examination of wood properties [1]. XETs probably play crucial roles in assimilating the products of photosynthesis into sugars and starch [48], in synthesizing cell wall biopolymers and in creating various glycosylated compounds [9]. Hence, we identified allelic polymorphisms in *PtoXET16A*, and examined their association with underlying growth and wood properties, using LD-based association in *P. tomentosa*. Our study identified 12 significant associations, which accounted for a small proportion of the phenotypic variance and were within the range of those published previously for forest trees [26]. Because of the low LD in *P. tomentosa* (Figure 4), once a marker-trait association has been discovered and validated, it is likely that such a marker is located in close proximity to the causal polymorphism or is the functional variant [49]. In total, eight of the 12 significant marker-trait associations representing six SNP markers were validated in at least one of the three subset populations. These SNPs, thus, provide powerful molecular markers for efficient marker-assisted breeding in *P*. *tomentosa*.

Of these, five SNP markers (SNP21, SNP22, SNP23, SNP27, and SNP29) associated with fiber length/width were observed using MLM, each explaining 3.40%–5.70% of the phenotypic variance (Table 3), supporting the idea that XET may have a strong effect on cell expansion in fibers [5]. Fibers, as the most abundant secondary wall-containing cells in the wood of dicot species, are affected by maturation (growth) stresses, particularly in tension wood [50]. One association between SNP15 and diameter at breast height was observed in the association population (Table 3) and validated in the subset populations. SNP15 is a missense mutation in exon 3 and results in an encoded amino acid change from Tyr to His. Many functional analyses of SNPs have examined coding regions in candidate genes related to wood traits, identifying SNPs that can alter proteins. These SNPs may have major effects on protein function, and thus on plant phenotype. Our study is supported by an analysis of *PtxtXET16-34* overexpression, in which xyloglucan distribution was higher in radial walls than in tangential walls, suggesting that *XETs* affect the expansion of radial walls especially during meristematic stage [5].

Intuitively, haplotypes may be more powerful than individual, non-ordered markers [35]. Of the markers identified in this study, SNP6, a missense mutation in exon 1, was significantly associated with lignin content and two haplotypes around SNP6 were also associated with lignin content traits (Table 3) suggesting that SNP6 may be a functional polymorphism that is in or near a locus that affects lignin content. Genotypic effect analysis showed that the trees heterozygous (GC) for this marker showed higher average lignin content than the homozygous trees (Figure 5), indicating over-dominance. Although there is no experimental evidence supporting a direct interaction, an indirect interaction between lignin and xyloglucan via connections with other wall components is possible. Pattathil *et al.* [51] suggested that lignin has a central role in overall wall structure, perhaps through direct covalent connections to diverse wall polysaccharides or through strong non-covalent interactions with these polymers. XETs, which are believed to be important regulators of cell wall expansion, specifically cutting the backbone of xyloglucan and re-forming a glycosidic bond with the free end of another xyloglucan chain [52].

Introns in a wide range of organisms including plants, animals, and fungi are able to increase the expression of the gene that they are contained in. In this study, we found a splicing site mutation, SNP16 (IVS3 + 2T>C) in intron3 of *PtoXET16A*. MLM testing indicated that it was closely associated with lignin content (*p* < 0.001, *FDR* < 0.05) (Table 3), suggesting that this splicing variant contributes to the variation in lignin content. SNPs in introns could affect phenotypic traits because those particular introns may play an important role in regulating gene expression [53]. Previous studies have demonstrated that introns can enhance eukaryotic gene expression and affect gene expression in diverse organisms including plants, insects, mice, and humans [54,55]. Some of this stimulation is due to a splicing-dependent increase in mRNA levels. To know if the expression level of alternatively spliced mRNA varied between the two groups (CT and TT), we used real-time PCR to quantify the mRNA levels in 30 trees, and observed significant differences in the RNA transcript levels in the association population. The mean relative expression levels of mRNA products for TT group and CT group were 1.4922 ± 0.4271 and 0.9175 ± 0.0828, respectively (Figure 7a). This indicated that this mutation affects the expression of *PtoXET16A*. Interestingly, in the association population, just two genotypes of SNP16 were observed (CT and TT). The number of genotypes was 391 and 34, respectively, suggesting that SNP16 may be a recessive lethal mutant (CC) or trees have a better wood phenotype with the T allele. Two haplotypes from SNP16-19 were associated with diameter at breast height and stem volume, indicating that the haplotypes containing the polymorphisms could cause variation in growth and wood quality traits.

## 4. Materials and Methods

### 4.1. Plant Materials

The association population consisted of 426 unrelated *P. tomentosa* individuals grown in Guan Xian County, Shandong Province, where root segments of 1047 native individuals collected from the entire natural distribution range of *P. tomentosa* were used to establish a clonal arboretum in 1982 using a randomized complete block design with three replications [56]. On the basis of principal component analysis and ISODATA fuzzy clustering of 16 meteorological factors [57], the total climatic zone covered by these individuals can be divided into three large climatic regions: southern, northwestern, and northeastern. In the present study, 426 unrelated individuals representing almost the entire geographic distribution of *P. tomentosa* (170 from the southern region, 91 from the northwestern region, and 165 from the northeastern region) were used for the SNP association analysis. In addition, a panel of 43 unrelated individuals (15 from the southern region, 14 from the northwestern region, and 14 from the northeastern region) was sequenced to identify SNPs within *PtoXET16A*.

### 4.2. Phenotypic Data

Phenotypic data of ten traits were measured, including lignin content, holocellulose content, alpha-cellulose content, fiber length, fiber width, microfibril angle, tree height, diameter at breast height, volume of wood, and tree height/tree diameter. The data of tree height and diameter at breast height were derived from field surveys in 2011 and used to evaluate the volume of wood. Wood cores were collected from each tree at a height 1.35 m above ground level, in which the variation in microfibril was characterized using an X-ray power diffractometer (Philips, Eindhoven, The Netherlands). These wood cores were then ground into wood meal, in which the holocellulose, α-cellulose, and lignin contents were determined using near-infrared reflectance spectroscopy according to Schimleck *et al.* [58]. Fiber length and width were measured using the Color CCTV Camera (Panasonic SD, Beijing, China). Furthermore, the SAS for Windows version 8.2 (SAS Institute, Cary, NC, USA) was used for ANOVA and correlation analysis for these phenotypic traits.

### 4.3. Isolation of PtoXET16A cDNA

The *P*. *tomentosa* cDNA library from xylem was constructed using the Superscript λ System according to the manufacturer’s instructions (Life Technologies, Rockville, MD, USA). The constructed cDNA library consisted of 5.0 × 10^6^ pfu with an insert size of 1.0–4.0 kb. Random end sequencing of 5000 cDNA clones and comparison with all available *XTH* sequences revealed that a full-length cDNA with high similarity to *PttXET16-34* was isolated and named *PtoXET16A*.

### 4.4. RNA Extraction

For RNA extraction, fresh tissue samples of root, leaf, and apex were collected from the 1-year-old *P. tomentosa* clone LM 50. The wood-forming tissues of upright stems, including developing and mature xylem tissues, were collected by scraping the thin (approximately 1.0 mm) and the deep layer on the exposed xylem surface at breast height; the other wood forming tissues, including phloem and cambium, were collected as described [59]. All tissues were immediately frozen in liquid nitrogen and stored at −80 °C. Total RNA was extracted from the various tissues using the Plant Qiagen RNAeasy kit (Qiagen, Shanghai, China) according to the manufacturer’s instructions. Additional on-column DNase digestions were performed three times during the RNA purification, using the RNase-Free DNase Set (Qiagen, Shanghai, China). RNA was then quantified and reverse transcribed into cDNA using the Superscript First-Strand Synthesis system and the supplied poly (T) primers (Invitrogen, Life Technologies, New York, NY, USA) [60].

### 4.5. Real-Time Quantitative PCR

Real-time quantitative PCR was performed on a DNA Engine Opticon 2 machine (MJ Research, Bio-Rad, Hercules, CA, USA) using the Light Cycler-Fast Star DNA master SYBR Green I kit (Roche, Basel, Switzerland). The *PtoXET16A*-specific and internal control (*Actin*) primer pairs were designed using Primer Express 3.0 software (Applied Biosystems, Life Technologies, New York, NY, USA). The PCR program included an initial denaturation at 94 °C for 5 min, and 40 cycles of 30 s at 94 °C, 30 s at 58 °C, and 30 s at 72 °C, and a final melt-curve of 70–95 °C. The specificity of the amplified fragments was checked by melting curve. All reactions were carried out in triplicate, and the data were analyzed using the Opticon Monitor Analysis Software 3.1 tool (MJ Research, Bio-Rad, Hercules, CA, USA).

### 4.6. Phylogenetic Analysis and Three-Dimensional Structures of PtoXET16A

To analyze the phylogenetic relationships between *PtoXET16A* and the *XTHs* from other species, the amino acid sequences of XTH family members, including the XTH family of *P. trichocarpa*, numbered according to Geisler-Lee *et al.* [9], *A.* *thaliana* XTH proteins, numbered according to Yokoyama and Nishitani [5]. *O. sativa* *XTH* gene products, numbered according to Yokoyama *et al.* [7], were identified by searching public databases available at NCBI (http://www.ncbi.nlm.nih.gov). Full-length protein sequences were used for the comparison and the gene models used are listed in Table S1. Phylogenetic analyses were conducted using MEGA version 4.0 (Arizona State University Campus, Phoenix, AZ, USA, 2007), and the neighbor-joining method was used to build phylogenetic trees [61]. Bootstrap analysis was performed using 1000 replicates. Three-dimensional structures of PtoXET16A and PttXET16-34 used Swissmodel (http://swissmodel.expasy.org/).

### 4.7. SNP Discovery and Genotyping

To identify SNPs within *PtoXET16A*, the entire gene was sequenced and analyzed in 43 unrelated individuals from the association population, without considering locations of insertions/deletions, using the software DnaSP4.90.1 (UB Web, Barcelona, Spain, 2010). All of these 43 sequences have been deposited in the GenBank database (GenBank Accession No. KM267487-KM267529). Thirty common SNPs were genotyped in 426 trees in the association population by amplification using locked nucleic acids [62,63]. Amplification was performed in a final reaction volume of 25 mL containing 20 ng genomic DNA, 0.8 U Taq DNA polymerase (Promega, Beijing, China), 50 ng forward primer, 50 ng reverse primer, 1× PCR buffer (Promega, Beijing, China), and 0.2 mM dNTPs (Promega, Beijing, China). The PCR conditions were: 94 °C for 3 min, 30 cycles of 94 °C denaturation for 30 s, annealing at 56–60 °C (depending on the primers) for 15 s, and extension at 72 °C for 1 min, with a final extension at 72 °C for 5 min.

### 4.8. Data Analysis

#### 4.8.1. Linkage Disequilibrium Analysis

To assess the pattern of linkage disequilibrium in the sequenced candidate gene region, the decay of LD with physical distance (base pairs) between SNP sites within *PtoXET16A* was estimated by linear regression analysis of linkage disequilibrium, using the DnaSP program version 4.90.1 (UB Web, Barcelona, Spain, 2010). The squared correlation of allele frequencies *r*^2^ [64] was used to test the LD between pairs of SNP markers using the software package HAPLOVIEW (http://www.broad.mit.edu/mpg/haploview.html). The interval value of the parameter varies from 0 to 1. The significance (*p*-values) of *r*^2^ for each SNP locus was calculated using 100,000 permutations.

#### 4.8.2. Association Testing

All trait-SNP association tests between 49 SNP markers and 10 traits were conducted using MLM with 10^4^ permutations in the software package TASSEL, version 2.0.1 (http://www.maizegenetics.net/) [65]. The MLM can be described as follows: *y* = μ + *Q*υ + *Zu* + *e*, where *y* is a vector of phenotype observation, μ is a vector of intercepts; υ is a vector of population effects; *u* is a vector of random polygene background effects; *e* is a vector of random experimental errors; *Q* is a matrix defining the population structure from STRUCTURE; and *Z* is a matrix relating *y* to *u*. Var (*u*) = G = σ^2^_a_κ with σ^2^_a_ as the unknown additive genetic variance and κ as the kinship matrix. In this Q + K model, the relative kinship matrix (K) was obtained using the method proposed by Ritland [66], which is built into the program SPAGeDi, Version 1.2 [67], and the population structure matrix (Q) was identified based on the significant subpopulations (K = 11) [68], as assessed according to the statistical model described by Evanno *et al.* [69], using 20 neutral genomic simple sequence repeat markers. The positive FDR method was applied to correct for multiple testing by using QVALUE software (University of Washington, Washington, DC, USA) [70].

#### 4.8.3. Haplotype Analysis

Haplotype frequencies from genotype data were estimated and haplotype association tests were done on a three-marker sliding window, using haplotype trend regression software. The significances of the haplotype associations were based on 1000 permutation tests. The modes of gene action were quantified using the ratio of dominant to additive effects estimated from least-square means for each genotypic class. Partial or complete dominance was defined as values in the range 0.50 < |d/a| < 1.25, whereas additive effects were defined as values in the range |d/a| ≤ 0.50. Values of |d/a| ≥ 1.25 were equated with under- or over-dominance. Details of the algorithm and formulas for calculating gene action were previously described [31,35].

## 5. Conclusions

In this study, we used association analysis to examine the relationship between polymorphisms in *PtoXET16A* and wood quality and yield in a *P. tomentosa* association population. Nucleotide diversity and LD patterns of *PtoXET16A* suggested differences between the geographical subsets *P. tomentosa* (Figure 4). We observed that nine SNPs, including two non-synonymous markers and one splicing variation, showed significant associations with growth and wood properties (Table 3). These SNPs explained a small proportion of the phenotypic variance, from 3.40% to 10.95%, consistent with a polygenic quantitative model. Haplotype analysis also indicated that the haplotypes containing the polymorphisms could cause variation in growth and wood quality traits. We also found that *PtoXET16A* is highly expressed in vascular tissues. Our study of *PtoXET16A* expression and genetic variation suggested that this XET may affect the expansion of radial walls. The SNP markers identified in this study can be used for marker-assisted selection to improve growth and wood-property traits in *P*. *tomentosa*.

## Supplemental Information

**Table S1:** *XTH* genes in *Arabidopsis thaliana*, *Oryza sativa* and *P. trichocarpa* utilizing in phylogenetic analysis.

Source	XTH No.	Genemodel
*Arabidopsis thaliana* ^a^	AtXTH4	AT2G06850
	AtXTH5	AT5G13870
	AtXTH8	AT1G11545
	AtXTH10	AT2G14620
	AtXTH17	AT1G65310
	AtXTH21	AT2G18800
	AtXTH27	AT2G01850
	AtXTH28	AT1G14720
	AtXTH30	AT1G32170
	AtXTH32	AT2G36870
	AtXTH33	AT1G10550
*Oryza sativa* ^b^	OsXTH2	AC136481
	OsXTH11	AP005445
	OsXTH23	AP005859
*P*.* trichocarpa* ^c^	PtXTH2	fgenesh4_pm.C_scaffold_131000027
	PtXTH3	grail3.0018003901
	PtXTH6	fgenesh4_pg.C_LG_VI000528
	PtXTH7	fgenesh4_pg.C_LG_IX001489
	PtXTH10	estExt_Genewise1Plus.C_LG_XI1472
	PtXTH11	estExt_Genewise1Plus.C_LG_XVIII2309
	PtXTH12	gw1.II.2740.1
	PtXTH13	gw1.V.599.1
	PtXTH14	eugene3.00140931
	PtXTH16	gw1.XI.1790.1
	PtXTH17	estExt_Genewise1Plus.C_LG_XVIII2321
	PtXTH18	gw1.XVIII.2856.1
	PtXTH19	gw1.XVIII.2837.1
	PtXTH20	fgenesh4_pg.C_scaffold_171000033
	PtXTH21	estExt_Genewise1_v1.C_LG_II3807
	PtXTH22	fgenesh4_pg.C_LG_IX000065
	PtXTH23	fgenesh4_pg.C_LG_XVIII000708
	PtXTH24	e_gw1.XI.2644.1
	PtXTH25	gw1.IV.1816.1
	PtXTH26	eugene3.00140870
	PtXTH27	estExt_Genewise1_v1.C_LG_I8040
	PtXTH28	e_gw1.V.604.1
	PtXTH29	eugene3.00090819
	PtXTH30	gw1.XIX.2748.1
	PtXTH31	estExt_fgenesh4_pg.C_LG_XVI0885
	PtXTH32	gw1.28.766.1
	PtXTH33	e_gw1.1681.2.1
	PtXTH34	estExt_Genewise1_v1.C_LG_III1278
	PtXTH35	e_gw1.XIX.2747.1
	PtXTH37	eugene3.00130049
	PtXTH38	estExt_Genewise1_v1.C_LG_XIV2162
	PtXTH39	estExt_Genewise1_v1.C_LG_VIII2102
	PtXTH40	grail3.0006015301
	PtXTH41	gw1.29.269.1

^a^ *Arabidopsis thaliana* XTH proteins, numbered according to Yokoyama and Nishitani [7]; ^b^ *Oryza sativa* *XTH* gene products, numbered according to Yokoyama *et al.* [8]; ^c^ *P. trichocarpa* *XTH* gene products, numbered according to Geisler-Lee *et al.* [9].
